# *Let’s Walk*: A Quasi-Experimental Multi-Component Intervention to Improve Physical Activity and Social Engagement for Older Chinese American Adults

**DOI:** 10.1007/s10903-024-01584-8

**Published:** 2024-02-13

**Authors:** Carina Katigbak, Ssu-Fang Cheng, Christina Matz, Holly Jimison

**Affiliations:** 1 Cizik School of Nursing, University of Texas Health Science Center at Houston, Houston, TX USA; 2 School of Public Health, University of Texas Health Science Center at Houston, Houston, TX USA; 3 School of Social Work, Boston College, Boston, MA USA; 4 Khoury College of Computer Sciences, Northeastern University, Boston, MA USA

**Keywords:** Chinese American, Older adults, Physical activity, Social engagement, Technology

## Abstract

Physical activity (PA) is critical for healthy aging, yet < 16% of U.S. older adults meet federal recommendations for moderate to vigorous PA. Asian Americans are a rapidly growing segment of the older adult population, who are less likely to meet these guidelines, and are frequently under-represented in clinical trials. This quasi-experimental pilot study evaluated the feasibility, acceptability, and preliminary effectiveness of a culturally tailored walking program to improve PA and social engagement for older Chinese Americans in Boston, MA. Participants at two community organizations were assigned to an enhanced walking or walking only condition for 12 weeks. Mixed effect repeated measures analysis addressed the study aims. The enhanced walking group (intervention) had fewer steps at baseline and less of a reduction in steps by 12 weeks as compared with the walking only (control) condition. Mean social engagement scores were significantly higher at 12 weeks (p = .03) for the intervention group. A culturally tailored walking intervention was feasible and acceptable for older Chinese Americans, improving social engagement and PA scores.

## Background

Physical activity (PA) is an important component of the American Heart Association’s, *Life’s Essential 8* [1]. An additional seven metrics consisting of a healthy diet, weight, sleep, healthy blood pressure, blood lipids, blood glucose, and avoiding nicotine comprise this construct of ideal cardiovascular health [1]. Current federal physical activity guidelines recommend that adults perform 150–300 min per week of moderate intensity exercise, such as brisk walking along with aerobic activity, muscle strengthening, bone-strengthening, flexibility, and balance activities [2]. The benefits of PA are well known and include a lower risk of cardiovascular disease, type 2 diabetes mellitus, certain cancers, and falls [3, 4] ​ In addition, consistent PA may lead to mental health benefits including a decreased risk of dementia and anxiety, and improved cognition and sleep [5, 6].

Despite these benefits, less than half of U.S. adults meet the federal recommendations for PA [2, 7]. Physical inactivity is linked to approximately $90 billion in annual healthcare costs [8], and accounts for 8.3% of premature mortality or deaths [9]. Ethnic minorities are disproportionally affected by CV disease [10] and are less likely to meet PA recommendations [11]. In particular, Asian Americans (AA) are a growing segment of the U.S. population [12] who are less likely to meet PA guidelines when compared to other racial groups [13, 14]. Lower acculturated AA are less likely to meet the physical activity guidelines [15], and this group may have significant needs for cultural and linguistic tailoring. Chinese Americans constitute the largest Asian ethnic subgroup in the U.S. [16, 17].

Our previous work examining PA among Asian American older adults identified specific contextual and cultural factors influencing PA engagement [18] and the need for interventions that are culturally and linguistically tailored to the target population [19]. A systematic review on the topic identified that those studies centered on Chinese Americans employed a variety of modalities including those that were education-focused [20] culturally tailored group classes held at YMCAs [21, 22], walking program [22], and Tai Chi [23]. Moreover, these studies lacked the inclusion of objective PA measures to validate self-reported PA [19]. In our formative qualitative work, we learned that Chinese older adults believe that Western exercises focused on repetitive movement (e.g. weight training) and physical exertion (e.g. running, jogging) are inconsistent with Eastern philosophies. Participants preferred activities described as “slow moving” and “calming” like TaiChi, QiGong, and walking - all of which are consistent with their cultural perspectives [18].

Thus, the *Let’s Walk* study overcame limitations in the literature through engaging participants in identifying acceptable PA modalities for this intervention, utilizing a culturally and linguistically appropriate communication platform, and incorporating objective measures of PA – a design element that was noticeably absent in previous work [19]. The pilot study aimed to test the preliminary effectiveness of a culturally and linguistically tailored walking-program with a cognitive behavioral component delivered via the Chinese social networking platform WeChat ™, to improve daily step counts and social engagement. *WeChat*™ is the largest mobile instant messaging communication tool and social networking service used among ethnic Chinese worldwide, with an estimated monthly user-base of > 880 million [24]. Compared to other available applications, WeChat’s ubiquitous presence among our local target population yielded exceptional interest and rapid study enrollment in our previous work [18, 25], and was thus adopted for this study since many older Chinese adults in the community were already using this platform. FitBit wrist-worn activity trackers were included to validate participants’ self-reports of physical activity; these devices are affordable, portable, and accurate in collecting physical activity data [26]. Moreover, the device’s step-count feature provides its users with feedback towards the need to increase or maintain their level of activity to meet their step-count goals.

## Theoretical Framework

Bandura’s Social Cognitive Theory [27] (SCT) served as the theoretical framework for this study. At the interpersonal level, SCT is used to facilitate behavior change. SCT suggests that the stronger one’s self-efficacy and outcome expectations, the more likely they are to initiate and persist with a given activity. Self-efficacy expectations are the individual’s beliefs in their capability to perform a course of action and achieve a desired outcome; and outcome expectations are the beliefs that a certain consequence will be produced by a personal action. Efficacy expectations are dynamic and enhanced by four mechanisms: (1) successful activity performance; (2) verbal persuasion; (3) seeing peers perform a similar activity; and (4) pleasant physiological and affective states associated with an activity [27]. Our approach to using WeChat and FitBit as tools to motivate participants’ physical activity addressed these domains as participants received encouraging prompts from the research team to engage in activity, interacted with each other on the platform to share their own progress towards reaching their physical activity goals, and provide peer support. Consistent with the theory, the objective feedback provided by the FitBit step count tracker served to promote participants to increase or maintain their physical activity, thereby increasing PA-related self-efficacy.

## Methods

### Participants

A power analysis was performed to estimate the statistical power under different scenarios of effect size and minimum detectable change in steps. A total of fifty subjects was required to detect an estimated effect size (δ) of 0.4044 and between subjects variance of 0.1090, with α = 0.05 and β = 0.80. To account for 10–15% attrition [28] and loss to follow up, we recruited 62 participants in partnership with community-based organizations serving older Chinese adults in Boston between March – June 2019. We used word-of-mouth advertising, posting flyers in the local community, online chat boards, and advertising in ethnic print media to reach our target population. In-person recruitment and informational events were held at scheduled gatherings of Chinese older adults at five Boston area community centers. Our community engaged research approach to building relationships with key community leaders, and increased visibility in Chinatown helped overcome well-known barriers to recruiting difficult to reach populations [29].

**Fig. 1:**
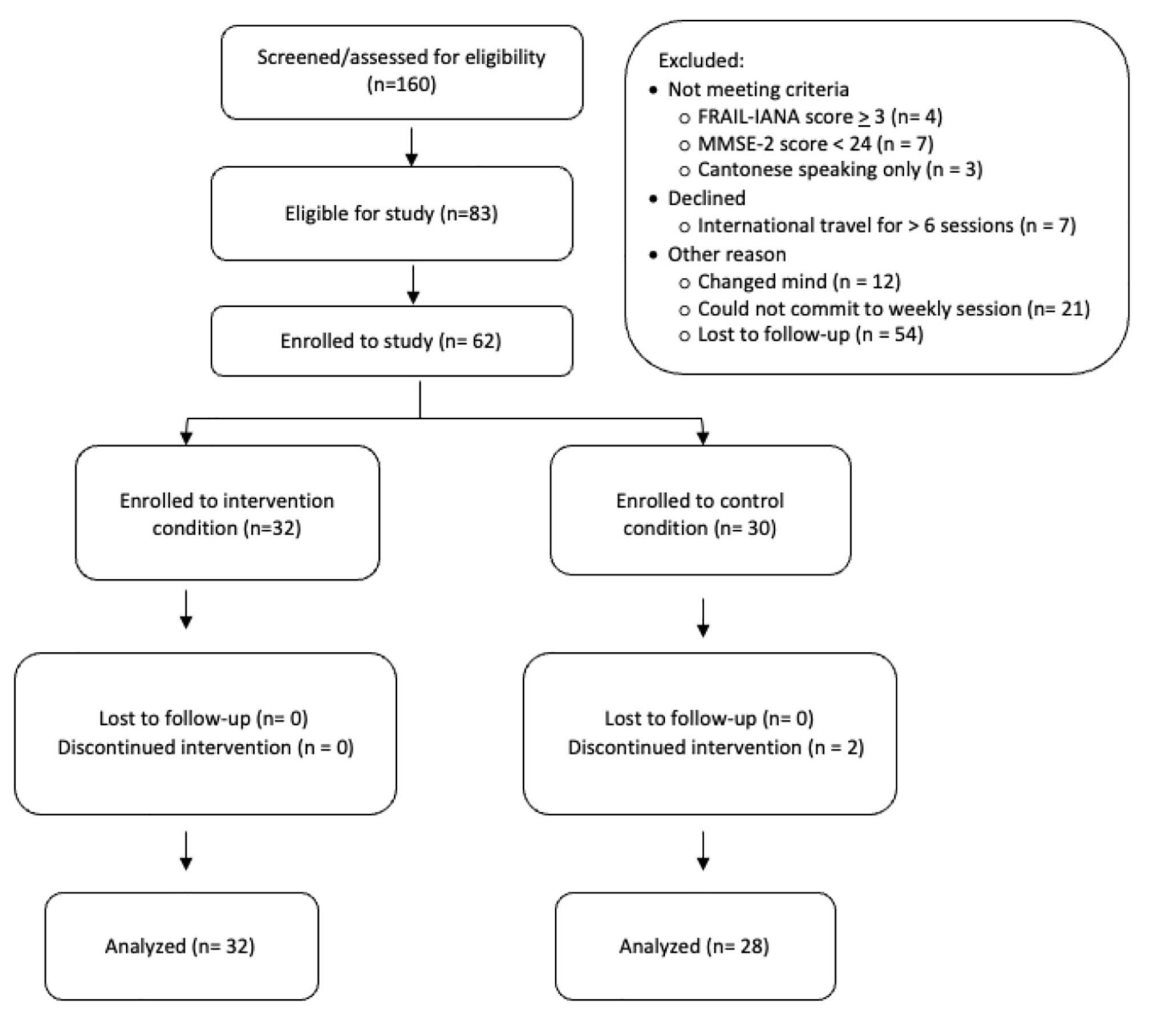
Study recruitment flowchart

A two –step screening process in Mandarin or English (per participant preference) assessed study eligibility which consisted of: community-dwelling older adults (≥ 60 years old), living in Boston, who self-identified as ethnically Chinese, communicated in Mandarin, had not fallen in the past 6 months, and owned an iOS or Android smartphone with data plan. A second in-person screening performed by trained research assistants with a health professions background (e.g., nursing, medical, and social work students) identified and excluded those with: (1) cognitive impairment (defined as a Mini-Mental Status Exam (MMSE-2) [30] score of < 24/30), and (2) frailty as determined by the FRAIL-IANA, Chinese version scores of ≥ 3) [31], (3) and those who did not speak Mandarin Chinese. A number of eligible participants (see Fig. 1). did not enroll to the study due to non-response at follow up, planned travel during the study period, loss of interest, and inability to commit to attending weekly sessions. A total of 62 participants met both screening criteria were enrolled to the study. At the study’s conclusion, participants kept the FitBit device and received a $50 Visa gift card in appreciation of their time. Ethics board approval was provided by the Boston College Institutional Review Board, protocol # 19.001.01.

### Data Collection

We employed a quasi-experimental non-randomized controlled design to test the preliminary effectiveness of an individualized walking program with group based weekly educational sessions on physical activity as measured through FitBit step tracker data and social engagement. Two Boston community-based organizations participating in an academic-community research partnership were respectively designated as the control (walking) or intervention (enhanced walking) arms. Surveys at baseline, 8 weeks, and 12 weeks follow up were administered via REDCap by bilingual (Mandarin and English) research assistants. We hypothesized that the intervention group would significantly increase their PA (e.g. higher step counts) and report greater degrees of social engagement at 8 and 12 weeks compared with the control condition.

Both groups received a FitBit Charge 3 to track daily steps, and attended six, 1-hour educational sessions on a variety of health promotion topics that included: healthy eating, activity for life, oral health, mental health, safety at home, and immunizations. These group based weekly in-person sessions allowed the research team to ensure that participant FitBit data were synced and uploaded to the study’s data collection platform, Fitabase. A two-week calibration period allowed participants to become familiar with the device, allowing the research team to collect baseline step-count data.

Based on this baseline step-count data, intervention group participants received individualized recommendations for a (5–15%) percentage increase in daily steps [32, 33]. This group was further divided into smaller *WeChat* groups (5 participants per group) moderated by a trained, bilingual research assistant (RA). Culturally appropriate motivational prompts such as “physical effort is vital for our bodies to function” and “daily, frequent physical movement keeps the illnesses and diseases away!” were informed by our formative work [18] and further refined with input from our community advisory board. These messages were theoretically aligned with House’s social support [34] constructs in the context of improving physical activity and were piloted with a test group, then sent through this platform 6 times per week throughout the 8-week study period. RAs monitored their respective *WeChat* groups and classified the social interactions taking place amongst study participants for future qualitative analysis.

### Measures

Eligibility screening measures assessed cognitive function through the validated Chinese version of the Mini-Mental Status Exam 2nd Edition (MMSE-2 ®) [30] to screen for dementia; participants were excluded if MMSE-2 ® <24/30, indicating abnormal cognitive function. Frailty was assessed with IANA-FRAIL [31], and scores > 3, indicating frailty, were a criterion for exclusion. We collected participant self-report demographic data; duration of residency in the the U.S. and English proficiency served as proxy measures for acculturation [35] and was assessed using a 3-item measure from the National Latino and Asian American Study (NLAAS) [36] and objectively measured physical activity (daily step counts) using the FitBit tracker. The International Physical Activity Questionnaire - Chinese version (IPAQ-C) [37] measured self-reported PA in occupational, household, and leisure activities over a one-week period. Social engagement was assessed using the Chinese version of Lubben’s Social Network Scale, a validated 12-item tool with scores ranging from 0 to 60, where higher scores indicate greater social engagement [38]. Finally, exercise self-efficacy [39] and self-rated health [40] were also assessed.

Feasibility was assessed by examining the percent of eligible participants that enrolled, along with the participants’ attendance and retention rates as they moved through the intervention. Acceptability was evaluated by asking participants to rate their satisfaction with the intervention on a 4-point Likert scale (1 = not satisfied and 4 = very satisfied).

### Analysis

All de-identified data were entered into SAS [version 9.4] software for management and analysis. Descriptive statistics were used to characterize the study sample. Differences in sociodemographic variables, health behaviors, and study outcomes by group assignment were assessed using the *x*^2^ test for categorical variables and the t-test for independent samples for continuous variables. Baseline assessment for number of steps was computed as the average number of steps over a two-week period prior to initiation of the intervention; post-assessment measures were taken over a 1-week period beginning 8 weeks after baseline, then again at 12 weeks. To evaluate our main outcomes of interest, linear mixed effect repeated measures analysis was conducted using number of steps and social engagement as the respective response variables at three time points (baseline, 8 weeks, and 12 weeks). Predictor variables were time (baseline, 8 weeks, and 12 weeks), treatment group, and their two-way interaction. Statistical significance was set at *p* < 0.05. Cohen’s d was used to estimate effect size for future studies.

## Results

The average age of study participants was 77 years, ranging from 63 to 92 years. Most participants were female (70%), married (62%), held a college degree (47%), an annual household income <$20,000 (80%), never used a physical activity tracker (87%), and had a mean English proficiency score (1.14 ± 0.27), indicating lower English-proficiency, and had lived in the U.S. an average of 20.7 years (± 12.7). As shown in Table 1, control and intervention groups did not significantly differ on any demographic characteristics.

**Table 1:** Demographic characteristics *n* = 60

Characteristic	Control Group	Intervention Group	*P*-value
	N (%)	N (%)	
Age (mean/SD)	75.14 ± 6.32	78.97 ± 4.48	0.32
Gender			0.24
Male	11 (39.29)	7 (21.88)	
Female	17 (60.71)	25 (78.12)	
Marital status			0.16
Single	5 (17.86)	1 (3.12)	
Married	16 (57.14)	21 (65.62)	
Widowed	6 (21.43)	10 (31.25)	
Separated	0	0	
Prefer not to answer	1 (3.57)	0	
Education			0.52
Less than high school	4 (14.29)	5 (15.62)	
Highschool	8 (28.57)	12 (37.50)	
Some college	0	1 (3.12)	
College degree	16 (57.14)	12 (37.50)	
Graduate degree or higher	0	1 (3.12)	
Prefer not to answer	0	1 (3.12)	
Income			0.23
≤ $20,000	20 (71.43)	28 (87.5)	
$20,000 -$29,999	1 (3.57)	0	
Prefer not to answer	7 (25.00)	4 (12.50)	
History of Chronic Illness			
Osteoporosis	20 (71.43)	20 (62.50)	0.55
Hypercholesterolemia	14 (50.00)	17 (53.12)	0.6
Hypertension	9 (32.14)	18 (56.25)	0.11
Type II Diabetes Mellitus	2 (7.14)	4 (12.50)	0.8
Heart Attack	2 (7.14)	0	0.09
Stroke	1 (3.57)	0	0.36
Renal Disease	1 (3.57)	1 (3.12)	0.3
Congestive Heart Failure	0	0	N/A
Previous physical activity tracker use			0.55
Yes No Prefer not to answer	4 (14.29) 24 (85.71) 0	3 (9.38) 28 (87.50) 1 (3.12)	
Years in the U.S. (mean/SD)	21.93 (± 13.5)	20.72 (± 13.4)	0.74
English proficiency (mean/SD)	1.17 (0.29)	1.11 (0.25)	0.47

**Table 2:** Study outcomes by group assignments *n* = 60

Outcome	Control Group	Intervention Group	*P*-Value
	Mean (± SD)	Mean (± SD)	
Step Count (FitBit data)			
Baseline	8747.35 (± 5319.38)	6568.76 (± 2920.38)	0.07
Week 8	10221.60 (± 5378.01)	7711.25 (± 2557.98)	0.04
Week 12	6848.37 (± 5385.61)	6812.58 (± 3507.98)	0.98
StudyArm*week			0.02
BMI			
Baseline	24.43 (± 3.10)	24.08 (± 3.40)	0.68
Week 8	N/A	N/A	N/A
Week 12	23.83 (± 3.06)	24.00 (± 2.95)	0.83
Social Engagement (LSNS)			
Baseline	44.58 (± 9.40)	45.20 (± 12.64)	0.87
Week 8	42.49 (± 9.42)	46.33 (± 8.26)	0.14
Week 12	42.17 (± 9.41)	47.70 (± 8.32)	0.03
StudyArm*week			0.28
Self-Efficacy for Exercise			
Baseline	46.64 (± 26.02)	51.48 (± 27.33)	0.49
Week 8	45.56 (± 20.54)	48.29 (± 20.18)	0.64
Week 12	53.81 (± 27.63)	60.15 (± 20.53)	0.34
IPAQ N(%)			
Baseline			
Low	1 (3.57)	1 (3.12)	0.93
Moderate	17 (60.71)	21 (65.62)	
High	10 (35.71)	10 (31.25)	
Week 8			
Low	2(8)	1(4)	0.74
Moderate	15(60)	14(56)	
High	8(32)	10(40)	
Week 12			
Low	0	1 (3.7)	0.27
Moderate	20 (74.07)	15 (55.56)	
High	7 (25.93)	11 (40.74)	

IPAQ = International Activity Questionnaire, LSNS = Lubben Social Network Scale

A total of 160 people were screened for the study, eighty-three were eligible to participate, and of these, 75% (*n* = 62) were enrolled to the study. Two people dropped out prior to session #2, unrelated to adverse events (attrition rate = 3%), for a final analytic sample to 60 participants, with 32 people enrolled to the intervention and 28 to the control group (Fig. 1). Attendance rates at group sessions were 74% and 81%, for the control and intervention groups, respectively. Missed sessions were not made up. With regards to acceptability, participants reported an overall satisfaction score of 3.62, on a scale of 1–4.

**Fig. 2:**
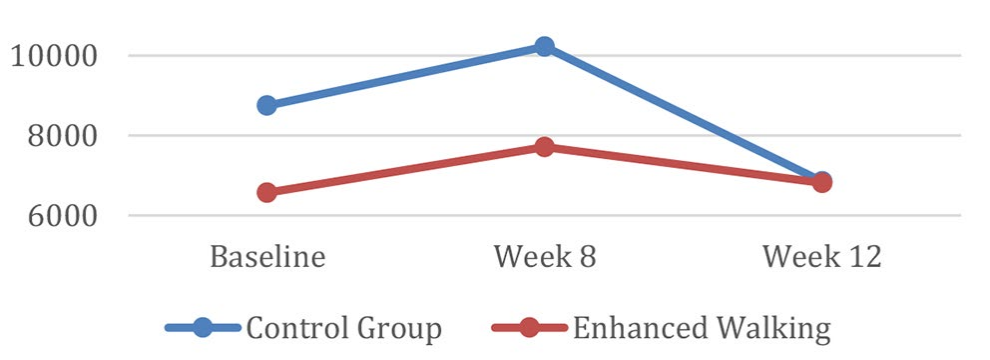
Step counts over time

Study outcomes by group assignment are provided in Table 2. Repeated measures analysis of the main effect of group assignment upon average step counts indicates a bell-shaped curvilinear relationship between step counts and time (see Fig. 2). Although baseline step counts were lower for the intervention group (6568 steps) compared to the control group (8747 steps), both groups’ post-intervention (week 8) step counts were reduced to approximately the same value by 12-week follow-up (intervention: 7711.25 to 6812 steps v. control: 10221.60 to 6848 steps).

Overall, the enhanced walking group improved their average daily step-counts from baseline to follow-up by 244 steps. In comparison, the control group’s average steps decreased by 1899 steps in this same timeframe. Finally, there was a significant (*p* = 0.02) two-way interaction between time and group assignment where the reduction in steps from post-intervention (week 8) to follow-up (week 12) was greater for the control group.

**Fig. 3:**
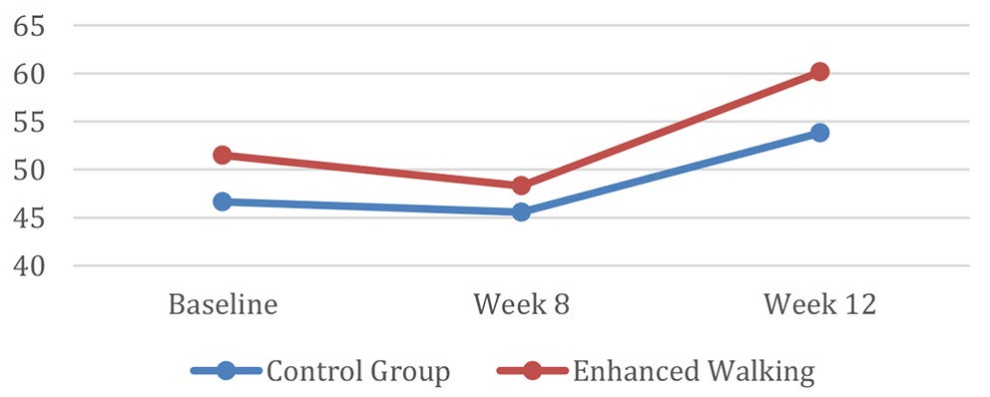
Social engagement over time

Repeated measures analysis for social engagement showed that mean scores were significantly higher (*p* = 0.03) at 12 weeks for the enhanced walking group (47.70 ± 8.31) vs. control group (42.17 ± 9.41). However, there was no significant interaction between time and group assignment for these mean social engagement (LSNS) scores (*p* = 0.28) (Fig. 3). Effect size (Cohen’s d) corresponding to change in step counts and social engagement were 0.73 and 0.38, respectively.

## Discussion

Culturally tailored interventions are critical to improving physical activity amongst ethnic minority, older adults. Differences in baseline step counts between the intervention group (6568 steps) and the control group (8747 steps) may not have accurately reflected the groups’ normal activities, but were rather a reflection of the initial “start-up” response to having their steps monitored. Consistent with findings from a recent meta-analysis [41, 42] the *Let’s Walk* trial found that initial improvements in physical activity and social engagement noted post-observation, were not maintained at follow-up. From post-intervention to follow-up, step counts declined by 899 steps for the intervention group, and 3,373 steps for the control group, resulting in a greater number of steps overall for the intervention group. It is possible that the multi-component approach to behavior change (e.g., individualized goal setting, feedback and monitoring, and social support) employed with the intervention group spurred these outcomes. However, the shift towards returning to baseline physical activity levels at follow-up aligns with research on physical activity maintenance [43], indicating that intervention effects diminish over time. The decline in PA amongst our intervention group – albeit less pronounced than the control group – may be explained by the abrupt withdrawal of supportive strategies provided through the intervention. Previous research on pedometer-driven physical activity interventions notes that in the absence of continued support and additional programming, initial increases in activity quickly degrade over time [44]. As part of our study design, frequent communications on WeChat, weekly group meetings, and the camaraderie of belonging to the group were intended to engage our participants. Gradually removing these elements or providing intermittent support from post-intervention to follow-up (week 8–12), as suggested by Kwasnicka and colleagues [45] could have been an effective strategy to mitigating the decline in activity and maintaining behavior change.

Social engagement scores from baseline to follow-up decreased for the control group and increased for the intervention group as hypothesized. In follow-up debriefing interviews with a subset of the intervention group, participants shared that they began arranging times to walk together during the week to make their activity pleasurable and enjoy time together. Recent work [46] proposes a synergistic relationship amongst physical activity interventions and improvements in social engagement. It is possible that individuals within the intervention group developed a sense of cohesion because of participating in a structured program. While this analysis was beyond the scope of this study, future work should seek to better understand how social engagement can be leveraged to improve physical activity long-term.

Engaging community members as partners in research development and implementation is critical to ensuring that interventions best meet their needs. We established credibility and rapport in forming a community academic research partnership years prior to *Let’s Walk*. We were intrigued to learn that the control group had a greater number of steps at baseline compared to the intervention group. This difference may be explained through the fact that the study site was situated in a densely populated area of the city, requiring greater travel by foot to execute daily activities. In contrast, the intervention group, while also within the city limits, was situated in a more car-dependent neighborhood, a characteristic that our study team did not fully consider during the study’s design-phase. Furthermore, differences in baseline step counts between the control and intervention group may be accounted for by the Hawthorne effect [47]. It is plausible that the knowledge that their step counts were being monitored, spurred a marked increase in step counts amongst the control group.

Limiting our assessment of acculturation to two proxy measures (e.g. English proficiency and duration of time living in the U.S.) overlooks the multidimensionality of the construct, and may not account for a number of other factors that may influence acculturation in this population [48]. Similarly, in our assessment of underlying chronic conditions, we did not include joint or rheumatological conditions that may hinder mobility and activity, and thus could have biased our sample of enrolled participants.

Study limitations relate to the quasi-experimental study design. Selection bias may have led to the inclusion of highly motivated individuals, which possibly explains the step count differences at 8 weeks between groups. Similarly, the characteristic difference between control and intervention sites in relation to neighborhood vehicle dependence may have further influenced our results. Randomization within each site and collecting baseline data before randomization may mitigate these differences in future studies. Concerns about diffusion of intervention within the sites were primary drivers to adopting our study approach. However, Handley and colleagues [49] highlight that quasi-experimental designs are appropriate for ‘real-world’ settings, given logistic challenges, particularly amongst groups that are difficult to access.

## Conclusion

Despite these limitations, our study contributes to the growing body of work providing insight to the value of culturally and linguistically adapted interventions for ethnic minority older adults. While our findings are aligned with others’ observations of initial improvements in physical activity that diminish over time, future work could examine what timepoints post-intervention are most sensitive to loss of intervention effect. Identifying these strategic points for additional support or intervention exposure would be helpful to maintaining behavior change.

Moving forward, it is imperative to address potential sources of bias in future studies. To mitigate the effects of selection bias and baseline characteristic differences between control and intervention groups, we recommend considering randomization within each site. Additionally, given the impact of neighborhood walkability on physical activity outcomes, future studies should explore the influence of contextual factors on the efficacy of interventions. By carefully considering these factors, we can develop more robust and generalizable interventions to improve physical activity among diverse populations.
